# Short Sleep Duration Among Middle School and High School Students — United States, 2015

**DOI:** 10.15585/mmwr.mm6703a1

**Published:** 2018-01-26

**Authors:** Anne G. Wheaton, Sherry Everett Jones, Adina C. Cooper, Janet B. Croft

**Affiliations:** ^1^Division of Population Health, National Center for Chronic Disease Prevention and Health Promotion, CDC; ^2^Division of Adolescent and School Health, National Center for HIV/AIDS, Viral Hepatitis, STD, and TB Prevention, CDC.

Insufficient sleep among children and adolescents is associated with increased risk for obesity, diabetes, injuries, poor mental health, attention and behavior problems, and poor academic performance (*1*–*4*). The American Academy of Sleep Medicine has recommended that, for optimal health, children aged 6–12 years should regularly sleep 9–12 hours per 24 hours and teens aged 13–18 years should sleep 8–10 hours per 24 hours (*1*). CDC analyzed data from the 2015 national, state, and large urban school district Youth Risk Behavior Surveys (YRBSs) to determine the prevalence of short sleep duration (<9 hours for children aged 6–12 years and <8 hours for teens aged 13–18 years) on school nights among middle school and high school students in the United States. In nine states that conducted the middle school YRBS and included a question about sleep duration in their questionnaire, the prevalence of short sleep duration among middle school students was 57.8%, with state-level estimates ranging from 50.2% (New Mexico) to 64.7% (Kentucky). The prevalence of short sleep duration among high school students in the national YRBS was 72.7%. State-level estimates of short sleep duration for the 30 states that conducted the high school YRBS and included a question about sleep duration in their questionnaire ranged from 61.8% (South Dakota) to 82.5% (West Virginia). The large percentage of middle school and high school students who do not get enough sleep on school nights suggests a need for promoting sleep health in schools and at home and delaying school start times to permit students adequate time for sleep.

The Youth Risk Behavior Surveillance System was designed to estimate the prevalence of health risk behaviors among students that contribute to the leading causes of death and disability in the United States at the national, state, territorial, tribal, and large urban school district levels.* Students complete an anonymous, voluntary, school-based paper-and-pencil questionnaire during a regular class period after the school obtains parental permission according to local procedures. The national high school YRBS is conducted by CDC. It uses a three-stage cluster sample design to obtain a nationally representative sample of students in public and private schools in grades 9–12 (*5*). In 2015, the student sample size was 15,624.^†^ The school and student response rates were 69% and 86%, respectively, resulting in an overall response rate of 60%.^§^

State and large urban school district high school and middle school surveys are conducted by health and education departments using a two-stage cluster sample designed to produce representative samples of students in each jurisdiction (*5*). These surveys are independent of CDC’s national YRBS and, unlike the national YRBS, are representative of only public school students, except in one state. To be included in this report, states and large urban school districts had to 1) have at least a 60% overall response rate, 2) include a question on sleep duration, and 3) provide permission for CDC to include their data. Thirty states and 16 large urban school districts administered a high school YRBS and met these criteria. Across these states, the student sample sizes ranged from 1,313 (South Dakota) to 55,596 (Maryland).^¶ ^The median overall response rate was 66.5% and ranged from 60% (Indiana and North Carolina) to 84% (Virginia). Across these large urban school districts, the high school student sample sizes ranged from 1,413 (Broward County, Florida) to 10,419 (District of Columbia). The median overall response rate was 76.5% and ranged from 64% (District of Columbia) to 88% (San Diego, California).

Nine states and seven large urban school districts administered a middle school YRBS and met these criteria. Across these states, the student sample sizes ranged from 1,640 (Kentucky) to 27,104 (Maryland). The median overall response rate was 76% and ranged from 68% (Maine) to 85% (Hawaii and Virginia). Across these large urban school districts, the middle school student sample sizes ranged from 1,333 (Los Angeles, California) to 4,533 (Duval County, Florida). The median overall response rate was 81% and ranged from 68% (San Francisco, California) to 86% (Orange County, Florida). All data sets were weighted to be representative of students in the jurisdiction.

All students in the national, state, and large urban school district surveys were asked to respond to this question about sleep duration: “On an average school night, how many hours of sleep do you get?” Possible responses were 4 or less hours, 5 hours, 6 hours, 7 hours, 8 hours, 9 hours, and 10 or more hours. Short sleep duration was defined as <9 hours for students aged 6–12 years and <8 hours for those aged 13–18 years. The analytic samples were composed of students who responded to both the sleep duration question and the age question.**

Prevalences and 95% confidence intervals (CIs) of short sleep duration on an average school night were calculated overall and by sex, grade, and race/ethnicity for the national high school YRBS and for a combined data set composed of data from the nine states that included the sleep duration question in a middle school YRBS. This combined data set is not nationally representative. The overall prevalence and 95% CI of short sleep duration also were calculated separately for each state and large urban school district at both middle school and high school levels. Pairwise differences in short sleep duration prevalence among sex, grade, and race/ethnicity subgroups were determined using t-tests; differences among estimates were considered statistically significant if the t-test p-value was <0.05. Analyses accounted for the weighting of the data and for the complex sampling designs.

The overall prevalence of short sleep duration among middle school students in the nine states combined was 57.8% (Table 1). The distribution of sleep duration was 5.9% for ≤4 hours, 6.0% for 5 hours, 11.0% for 6 hours, 20.0% for 7 hours, 29.9% for 8 hours, 17.2% for 9 hours, and 10.0% for ≥10 hours. The prevalence of short sleep duration in this combined sample was higher among female students (59.6%) than among male students (56.0%). The prevalence of short sleep duration also was highest among students in grade 6 (61.3%), lowest among students in grade 8 (53.1%), and higher among black (61.1%) and Native Hawaiian/Pacific Islander (64.2%) students than among white (56.6%), Hispanic (57.3%), and Asian (55.5%) students. State-specific estimates of short sleep duration ranged from 50.2% (New Mexico) to 64.7% (Kentucky). Prevalence estimates for the seven large urban school districts ranged from 50.2% (San Francisco, California) to 61.8% (Miami-Dade County, Florida).

**TABLE 1:** Prevalence of short sleep duration* on an average school night among middle school students in nine states combined and among nine states and seven large urban school districts, by selected characteristics — Youth Risk Behavior Surveys, 2015

Site/Characteristic	No.^†^	Prevalence % (95% CI)
Nine state surveys combined^§^	52,356	57.8 (56.7–58.9)
**Sex**		
Female	26,549	59.6 (58.2–61.0)^¶^
Male	25,608	56.0 (54.6–57.4)^¶^
**Grade**		
6	14,060	61.3 (59.5–63.0)**^,††^
7	19,153	59.2 (57.8–60.5)^§§,††^
8	18,707	53.1 (51.6–54.7)^§§,^**
**Race/Ethnicity**		
White^¶¶^	23,434	56.6 (54.9–58.4)***^,†††^
Black^¶¶^	7,638	61.1 (59.0–63.1)^§§§,¶¶¶,^****
Hispanic	8,384	57.3 (55.3–59.3)***^,†††^
Asian^¶¶^	2,644	55.5 (51.0–59.8)***^,†††^
American Indian/Alaska Native^¶¶^	1,302	59.4 (55.3–63.4)
Native Hawaiian/Pacific Islander^¶¶^	2,075	64.2 (59.1–68.9)^§§§,¶¶¶,^****
**State surveys**		
Delaware	2,883	58.8 (56.7–60.9)
Florida	5,472	56.9 (54.9–58.9)
Hawaii	5,704	61.3 (57.4–65.0)
Kentucky	1,603	64.7 (61.7–67.5)
Maine	4,852	53.0 (50.8–55.1)
Maryland	24,938	58.7 (57.5–59.9)
New Mexico	2,961	50.2 (48.2–52.3)
Virginia	2,133	56.3 (53.7–58.9)
West Virginia	1,810	64.1 (60.7–67.4)
**Large urban school district surveys**		
Broward County, Florida	1,447	62.0 (58.7–65.2)
Duval County, Florida	4,259	58.5 (56.7–60.2)
Houston, Texas	2,326	58.3 (55.5–60.9)
Los Angeles, California	1,223	54.2 (50.8–57.5)
Miami-Dade County, Florida	2,129	61.8 (58.9–64.6)
Orange County, Florida	1,799	53.1 (50.4–55.8)
San Francisco, California	1,861	50.2 (47.0–53.4)

**Abbreviation:** CI = confidence interval.

* Short sleep duration defined as <9 hours for students aged 6–12 years and <8 hours for students aged 13–18 years.

^†^ Unweighted number of survey respondents. Categories might not sum to sample total because of missing responses.

^§^ A combined data set using data from nine state surveys (Delaware, Florida, Hawaii, Kentucky, Maine, Maryland, New Mexico, Virginia, and West Virginia) that is not nationally representative.

^¶^ Significantly different by sex (p<0.05).

** Significantly different from grade 7 (p<0.05).

^††^ Significantly different from grade 8 (p<0.05).

^§§^ Significantly different from grade 6 (p<0.05).

^¶¶^ Non-Hispanic.

*** Significantly different from black students (p<0.05).

^†††^ Significantly different from Native Hawaiian/Pacific Islander students (p<0.05).

^§§§^ Significantly different from white students (p<0.05).

^¶¶¶^ Significantly different from Hispanic students (p<0.05).

**** Significantly different from Asian students (p<0.05).

At the high school level, nationwide, the prevalence of short sleep duration was 72.7% (Table 2). The distribution of sleep duration was 7.5% for ≤4 hours, 12.6% for 5 hours, 22.9% for 6 hours, 29.7% for 7 hours, 20.6% for 8 hours, 5.0% for 9 hours, and 1.7% for ≥10 hours. The prevalence of short sleep duration was higher among female students (75.6%) than among male students (69.9%), lower among students in grade 9 (65.6%) than in other grades (71.7%–77.6%), and higher among black (76.5%) and Asian (79.3%) students than white (72.0%) and Hispanic (70.2%) students. State-level estimates of short sleep duration for the 30 states ranged from 61.8% (South Dakota) to 82.5% (West Virginia) (Table 2) (Figure). Prevalence estimates for the 16 large urban school districts ranged from 69.9% (Los Angeles, California) to 85.6% (Broward County, Florida).

**TABLE 2:** Prevalence of short sleep duration* on an average school night among high school students, nationwide and among 30 states and 16 large urban school districts, by selected characteristics — Youth Risk Behavior Surveys, 2015

Site/Characteristic	No.^†^	Prevalence % (95% CI)
National survey	14,471	72.7 (70.4–74.9)
**Sex**		
Female	7,250	75.6 (73.3–77.7)^§^
Male	7,165	69.9 (66.9–72.7)^§^
**Grade**		
9	3,673	65.6 (62.6–68.5)^¶,^**^,††^
10	3,593	71.7 (69.2–74.0)^§§,^**^,††^
11	3,695	77.1 (73.5–80.3)^§§,¶^
12	3,426	77.6 (74.7–80.2)^§§,¶^
**Race/Ethnicity**		
White^¶¶^	6,592	72.0 (69.5–74.4)***^,†††^
Black^¶¶^	1,381	76.5 (72.8–79.9)^§§§,¶¶¶^
Hispanic	4,729	70.2 (66.6–73.5)***^,†††^
Asian^¶¶^	606	79.3 (72.2–85.0)^§§§,¶¶¶^
American Indian/Alaska Native^¶¶^	150	75.0 (60.0–85.7)
Native Hawaiian/Pacific Islande^¶¶^	86	—****
**State surveys**		
Alabama	1,505	72.0 (69.1–74.7)
Arkansas	2,656	70.7 (66.3–74.7)
California	1,894	71.0 (65.1–76.3)
Connecticut	2,167	80.1 (78.3–81.9)
Delaware	2,503	75.7 (73.1–78.1)
Florida	6,057	76.9 (75.4–78.3)
Hawaii	5,528	75.3 (72.7–77.8)
Illinois	3,043	76.7 (73.9–79.3)
Indiana	1,871	78.6 (76.2–80.8)
Kentucky	2,495	75.7 (72.7–78.5)
Maryland	52,043	76.2 (75.5–76.9)
Massachusetts	3,015	78.0 (75.7–80.2)
Michigan	4,717	79.8 (77.1–82.2)
Missouri	1,432	72.6 (69.0–75.9)
Montana	4,371	67.4 (65.6–69.2)
Nebraska	1,449	68.1 (64.5–71.4)
Nevada	1,393	75.9 (73.2–78.4)
New Hampshire	13,903	71.6 (70.1–73.1)
New Mexico	7,787	68.3 (66.7–69.8)
New York	8,129	78.1 (75.8–80.3)
North Carolina	5,683	75.0 (71.4–78.3)
North Dakota	2,094	70.5 (67.8–73.0)
Oklahoma	1,586	71.8 (68.5–74.9)
Pennsylvania	2,715	74.3 (71.9–76.6)
South Carolina	1,272	72.1 (68.0–75.8)
South Dakota	1,296	61.8 (57.6–65.8)
Tennessee	4,015	70.7 (69.1–72.2)
Virginia	4,264	72.8 (70.4–75.1)
West Virginia	1,561	82.5 (79.2–85.3)
Wyoming	2,328	69.8 (67.7–71.7)
**Large urban school district surveys**		
Boston, Massachusetts	1,547	82.4 (79.8–84.7)
Broward County, Florida	1,327	85.6 (83.3–87.6)
Cleveland, Ohio	1,434	80.0 (77.7–82.1)
DeKalb County, Georgia	1,814	80.4 (78.3–82.5)
District of Columbia	10,281	71.6 (70.5–72.7)
Duval County, Florida	3,153	81.1 (79.1–83.0)
Houston, Texas	2,878	75.6 (73.5–77.6)
Los Angeles, California	2,189	69.9 (66.3–73.3)
Miami-Dade County, Florida	2,629	80.4 (77.9–82.7)
New York City, New York	5,972	74.8 (72.4–77.1)
Oakland, California	1,512	70.6 (66.7–74.2)
Orange County, Florida	1,421	79.3 (76.2–82.1)
Palm Beach, Florida	2,284	81.5 (79.2–83.6)
Philadelphia, Pennsylvania	1,464	80.3 (77.1–83.2)
San Diego, California	2,249	71.9 (68.8–74.9)
San Francisco, California	2,005	75.2 (72.3–77.9)

**Abbreviation:** CI = confidence interval.

* Short sleep duration defined as <9 hours for students aged 6–12 years and <8 hours for students aged 13–18 years.

^†^ Unweighted number of survey respondents. Categories might not sum to sample total because of missing responses.

^§^ Significantly different by sex (p<0.05).

^¶^ Significantly different from grade 10 (p<0.05).

** Significantly different from grade 11 (p<0.05).

^††^ Significantly different from grade 12 (p<0.05).

^§§^ Significantly different from grade 9 (p<0.05).

^¶¶^ Non-Hispanic.

*** Significantly different from black students (p<0.05).

^†††^ Significantly different from Asian students (p<0.05).

^§§§^ Significantly different from white students (p<0.05).

^¶¶¶^ Significantly different from Hispanic students (p<0.05).

**** Unreliable estimate. Denominator <100 students.

**FIGURE:**
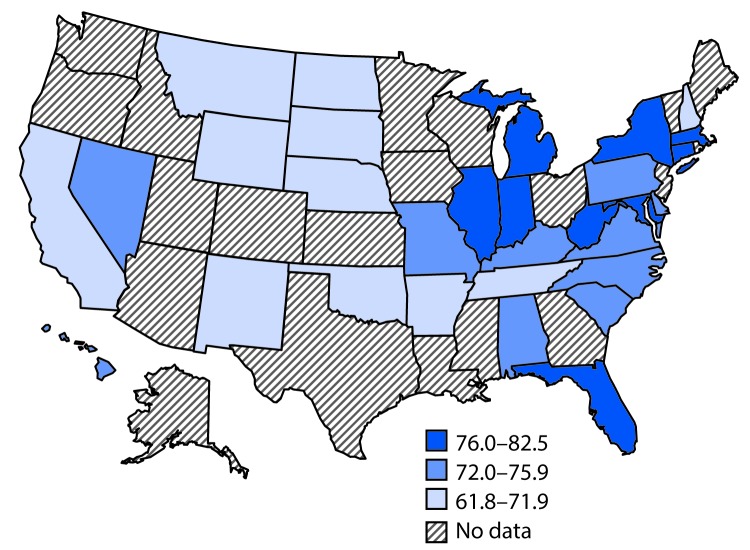
Prevalence of short sleep duration* on an average school night among high school students, by state — Youth Risk Behavior Survey, 2015 * Short sleep duration defined as <9 hours for students aged 6–12 years and <8 hours for students aged 13–18 years.

## Discussion

Children and adolescents who do not get the recommended amount of sleep for their age are at increased risk for chronic conditions such as diabetes, obesity, and poor mental health, as well as injuries, attention and behavioral problems, and poor academic performance (*1*–*4*). In addition, short sleep duration has been found to be associated with engaging in health- and injury-related risk behaviors among high school students (*6*,*7*). The national high school YRBS has included a question about sleep duration since 2007, and it is used to track the progress of the *Healthy People 2020* sleep objective for this population (Sleep Health Objective 3: Increase the proportion of students in grades 9 through 12 who get sufficient sleep).^††^ Nationally, no progress has been made toward this objective: the percentage of high school students who get sufficient sleep has substantially decreased from 30.9% in 2009, the baseline year for this objective, to 27.3% in 2015, the latest year of available data.^§§^ A question about sleep duration was included for the first time in 2015 in the standard middle school and high school YRBS questionnaires used as the starting point for the state and large urban school district YRBS questionnaires. As a result, evidence now exists that short sleep duration is prevalent among middle school students as well as high school students. In addition, at both middle and high school levels, in every state and large urban school district with YRBS data about sleep duration, a majority of students reported getting less than the recommended amount of sleep.

The findings in this report are subject to at least four limitations. First, sleep duration was obtained by self-report and was not verified by objective measures such as actigraphy (sensor measurement of motor activity) or polysomnography (sleep study). Second, a national YRBS is not conducted among middle school students. The middle school findings from the combined data set cannot be generalized to the entire United States. Third, at both middle and high school levels, state-level data are not available for states that did not administer the YRBS, did not include a question about sleep duration on their YRBS, or did not achieve a high enough overall response rate to obtain weighted data. Finally, YRBS data are representative only of students enrolled in school; in 2015, less than 5% of children aged 7–17 years were not enrolled in school.^¶¶^

To ensure their children get enough sleep, parents can support the practice of good sleep habits. One important habit is maintaining a consistent sleep schedule during the school week and weekends. Parent-set bedtimes have been linked to getting enough sleep among adolescents (*8*). Evening light exposure and technology use are also associated with less sleep among adolescents (*9*). Parents can limit children’s permitted use of electronic devices in terms of time (e.g., only before a specific time, sometimes referred to as a “media curfew”) and place (e.g., not in their child’s bedroom) Other tips for better sleep are available at https://www.cdc.gov/sleep/about_sleep/sleep_hygiene.html. One meta-analysis of school-based sleep education programs found that they produced significantly longer weekday and weekend total sleep time immediately after completion, but that these improvements were not maintained at follow-up (*10*). Researchers designing such programs might consider incorporating refresher sessions to maintain improvements in sleep duration and sleep hygiene (i.e., habits that support good sleep). School districts can also support adequate sleep among students by implementing delayed school start times as recommended by the American Academy of Pediatrics,*** the American Medical Association,^†††^ and the American Academy of Sleep Medicine.^§§§^

**Summary**

**What is already known about this topic?**

Insufficient sleep among children and adolescents is associated with an increased risk for obesity, diabetes, injuries, poor mental health, attention and behavior problems, and poor academic performance. Nationwide, approximately two thirds of U.S. high school students report sleeping <8 hours per night on school nights.

**What is added by this report?**

This is the first report to provide state-level estimates of short sleep duration among middle school and high school students using age-specific recommendations from the American Academy of Sleep Medicine. A majority of both middle school and high school students in states and large urban school districts included in this report get less than the recommended amount of sleep, putting them at an increased risk for several chronic conditions.

**What are the implications for public health practice?**

The finding that a large percentage of middle school and high school students who do not get enough sleep on school nights provides an opportunity for promoting sleep health in schools, including addition of sleep health to curricula and delaying school start times to permit students adequate time for sleep.

## Footnotes

* https://www.cdc.gov/healthyyouth/data/yrbs/overview.htm.

† https://www.cdc.gov/healthyyouth/data/yrbs/pdf/2015/ss6506_updated.pdf.

§ Overall response rate = school response rate x student response rate ([number of participating schools/number of eligible sampled schools] x [number of usable questionnaires/number of eligible students sampled]).
